# The lysosome as a novel therapeutic target of EGFR-mediated tumor inflammation

**DOI:** 10.3389/fphar.2022.1050758

**Published:** 2022-11-11

**Authors:** Woo Jung Sung, Dohyang Kim, Anlin Zhu, Namki Cho, Hee Min Yoo, Ji Heon Noh, Kyoung Mi Kim, Hyun-Su Lee, Jaewoo Hong

**Affiliations:** ^1^ Department of Pathology, Daegu Catholic University School of Medicine, Daegu, South Korea; ^2^ Department of Physiology, Daegu Catholic University School of Medicine, Daegu, South Korea; ^3^ College of Pharmacy, Chonnam National University, Gwangju, South Korea; ^4^ Biometrology Group, Korea Research Institute of Standards and Science, Daejeon, South Korea; ^5^ Department of Precision Measurement, University of Science & Technology (UST), Daejeon, South Korea; ^6^ Department of Biochemistry, Chungnam National University, Daejeon, South Korea; ^7^ Department of Biological Sciences, Chungnam National University, Daejeon, South Korea

**Keywords:** ErbB, EGFR, TKI, lysosome, combination therapy

## Abstract

EGFR-mediated tumors have been targeted to overcome several different malignant cancers. EGFR overexpression and mutations are directly related to the malignancy, which makes the therapy more complicated. One reason for the malignancy is the induction of AP1 followed by inflammation *via* IL-6 secretion. Current therapeutic strategies to overcome EGFR-mediated tumors are tyrosine kinase inhibitors (TKIs), anti-EGFR monoclonal antibodies, and the combination of these two agents with classic chemotherapy or immune checkpoint inhibitors (ICIs). Although the strategies are straightforward and have shown promising efficacy in several studies, there are still hurdles to overcoming the adverse effects and limited efficacy. This study reviews the current therapeutic strategies to target EGFR family members, how they work, and their effects and limitations. We also suggest developing novel strategies to target EGFR-mediated tumors in a novel approach. A lysosome is the main custodial staff to discard unwanted amounts of EGFR and other receptor tyrosine kinase molecules. Targeting this organelle may be a new approach to overcoming EGFR-mediated cancers.

## 1 Introduction

Lysosomes are acidic intracellular organelles carrying a package of hydrolytic enzymes and various membrane-associated proteins (Saftig, 2005). Lysosomes exist in all animal cells except erythrocytes, but the structure and number differ depending on the cell type and functions (Saftig, 2005; Appelqvist et al., 2013). The characteristic highly acidic pH (4.5–5.0) of lysosomes is achieved by the proton pump of endosomes, the vacuolar-type H^+^-ATPase (V-ATPase). The acidic environment of lysosomes enables the activity of hydrolases optimized in acidic pH (Saftig, 2005). The biogenesis of lysosomes is known to be controlled by the coordination of the regulatory gene network and the lysosomal expression, which is governed by the nuclear translocation of transcription factor EB (TFEB) (Sardiello et al., 2009; Palmieri et al., 2011; Settembre et al., 2012).

Lysosomal gene mutations can lead to reduced production or mislocalization of lysosomal enzymes and cell waste accumulation. The importance of lysosomal dysfunctions is underestimated. In cancers, nascent biomass production is required for cellular transformation, and lysosomal function has a vital role in macromolecular synthesis by precursors (Davidson and Vander Heiden, 2017). The other importance of lysosomes is the adaptation to nutrient stress, which is the further provocation by cancers, and autophagy is known to play an essential role in some tumor progressions (Levine and Kroemer, 2008; Kimmelman, 2011; White, 2015). Lysosomal enzyme activity is known to be increased in many tumors compared to neighboring normal tissue, and the lysosomal changes in cancers have been known for tens of years (Kirkegaard and Jaattela, 2009). Many cancer hallmarks lead to or are resulted from lysosomal functioning or malfunctioning (Hanahan and Weinberg, 2011). Additionally, a recent insight has suggested that the increase in lysosomal activity can lead to the downregulation of receptor tyrosine kinase activity in cancer cells (Hong et al., 2019).

Receptor tyrosine kinase (RTK) activity is well-known for the over-expression and over-activation in many cancer cells. Their implications of cancer malignancy in proliferation, metastasis, angiogenesis, and invasion have been studied for decades. RTKs are tightly related to the over-expression and exacerbated activation in cancer cells (Toulany, 2019). The epidermal growth factor receptor (EGFR) is a member of the ErbB family of RTKs and has critical roles in epithelial physiology (Schlessinger, 2014). Mutations and overexpression of EGFR in various human cancers are frequent; hence, EGFR targets several current cancer therapies (Yarden and Pines, 2012).

The EGFR pathway has been studied since the 1980s following the discovery of EGF in the 1960s. The biochemical, structural, and genetic studies of EGFR revealed the molecular mechanism of the ligand-mediated receptor transphosphorylation and serial activation of the intracellular signaling consequences. The activation of the EGFR pathway induces multiple cell proliferation pathways, survival, and differentiation from the cell surface to the nucleus through the intracellular endosomal system (Lemmon and Schlessinger, 2010; Schlessinger, 2014). The canonical EGFR pathway is activated in a ligand- and kinase-dependent manner (Lemmon and Schlessinger, 2010). Additionally, a kinase-independent pathway has been identified, which showed unexpected roles regulating autophagy and metabolism (Tan et al., 2016). The stress signals usually induce the noncanonical pathway of EGFR, and the stress signals can be easily observed in cancer cells providing survival and resistance to therapies (Jutten et al., 2013; Tan et al., 2016). These findings lead to the idea that targeting both kinase-dependent and independent pathways of EGFR can offer an additional opportunity in cancer therapies. This review aims to understand how lysosomal changes can affect EGFR-mediated cancers and suggest a new approach to developing cancer therapies targeting lysosomes and EGFR.

## 2 EGFR (ErbB) family members

EGFR family members are expressed in various cell types, such as epithelial, mesenchymal, and neuronal cells, where they show diverse roles in development, proliferation, and differentiation (Casalini et al., 2004). All four EGFR family members are RTKs. They have a highly homogeneous structure consisting of an extracellular domain for ligand binding, a hydrophobic transmembrane region, and an intracellular region for signal transduction with a conserved tyrosine kinase domain (Prenzel et al., 2001). EGFR family members bind with a ligand family of twelve polypeptides, followed by the stimulation of the homodimeric or heterodimeric interactions between family members. The interaction leads to the autophosphorylation of several intracellular tyrosine residues (Roskoski, 2004). When the tyrosine residue is phosphorylated, multiple adaptors and signaling molecules are docked to the phosphorylated site that, generate diverse responses (Hynes et al., 2001). EGFR family members are involved in several signaling pathways, such as the PI3K-Akt and RAS-ERK. EGFR (ErbB1) and ErbB4 are canonical RTK molecules interacting and signaling by specific ligands. However, ErbB2 (HER2) does not interact with any known ligand, and ErbB3 is generally accepted as missing kinase activity (Hynes et al., 2001).

Interestingly, ErbB3 has been recently known to interact with ATP inducing a low level of kinase activity (Shi et al., 2010). Because ErbB2 and ErbB3 lack the classic characteristics of EGFR family receptors, these receptors form a heterodimer with other family members. Heterodimerization of these members signals diversity and amplifies the response among EGFR family members. Among the dimers, ErbB2-ErbB3 heterodimers produce the most potent mitogenic signals (Zhang et al., 2012).

## 3 EGFR family overexpression and tumor inflammation

Uncontrolled activity of the EGFR family can lead to aberrant stimulation of growth and tumorigenesis in many tumor types, such as lung, brain, breast, colon, and head and neck tumors (Yarden and Pines, 2012). Abnormal EGFR and ErbB2 activation can be observed in a wide range of mechanisms like overexpression, gene mutations, and autocrinergic stimulation in cancers (Hynes and MacDonald, 2009). When EGFR and ErbB2 are mutated or overexpressed, receptors can be activated regardless of ligan-binding. In addition, these receptors show higher activity than usual with ligand binding. As stated above, ErbB3 has low kinase activity, so the oncogenic activity is observed mostly when dimerized with EGFR or ErbB2 (Hynes and MacDonald, 2009). Erb4 is a classic RTK but has multiple variants showing different activities. Some of the variants are oncogenic, while others are tumor-suppressive (Sundvall et al., 2008). However, a recent report showed that ErbB4 point mutations exist at low levels in several types of tumors, which implies that ErbB4 activation is highly related to pro-tumorigenicity (Prickett et al., 2009).

The hyperactivation of EGFR family members is not uncommon in several cancers, making EGFRs an attractive therapeutic target. Among four members of the EGFR family members, EGFR and ErbB2 are best studied and known for their roles in cancer, so most drugs developed and under clinical trials are targeting these two receptors. Most strategies targeting EGFR and ErbB2 are blocking the ligand and receptor binding or inhibiting the kinase activity of receptors. However, the combination of several antibodies or kinase inhibitors showed promising additive and synergetic therapeutic effects in studies (Tebbutt et al., 2013). In this study, we want to review and discuss new therapeutic strategies targeting EGFR families in aspects of ligand-binding, kinase, and else.

## 4 EGFR and inflammation

Immune cells infiltrating the tumor are not the only producers of proinflammatory cytokines but also tumor cells over-express cytokines to gather leukocytes to the tumor site. The cytokine-releasing tumor cells produce a cocktail of cytokines, such as interleukins and interferons, to maintain the tumor microenvironment to continue oncogenic signalings (Crusz and Balkwill, 2015). IL-1, IL-6, IL-11, IL-15, IL-17, IL-23, and TNF-α are representative proinflammatory cytokines promoting tumor cell differentiation, proliferation, and survival that build up the tumor microenvironment and tumor inflammation (Vendramini-Costa and Carvalho, 2012).

**FIGURE 1:**
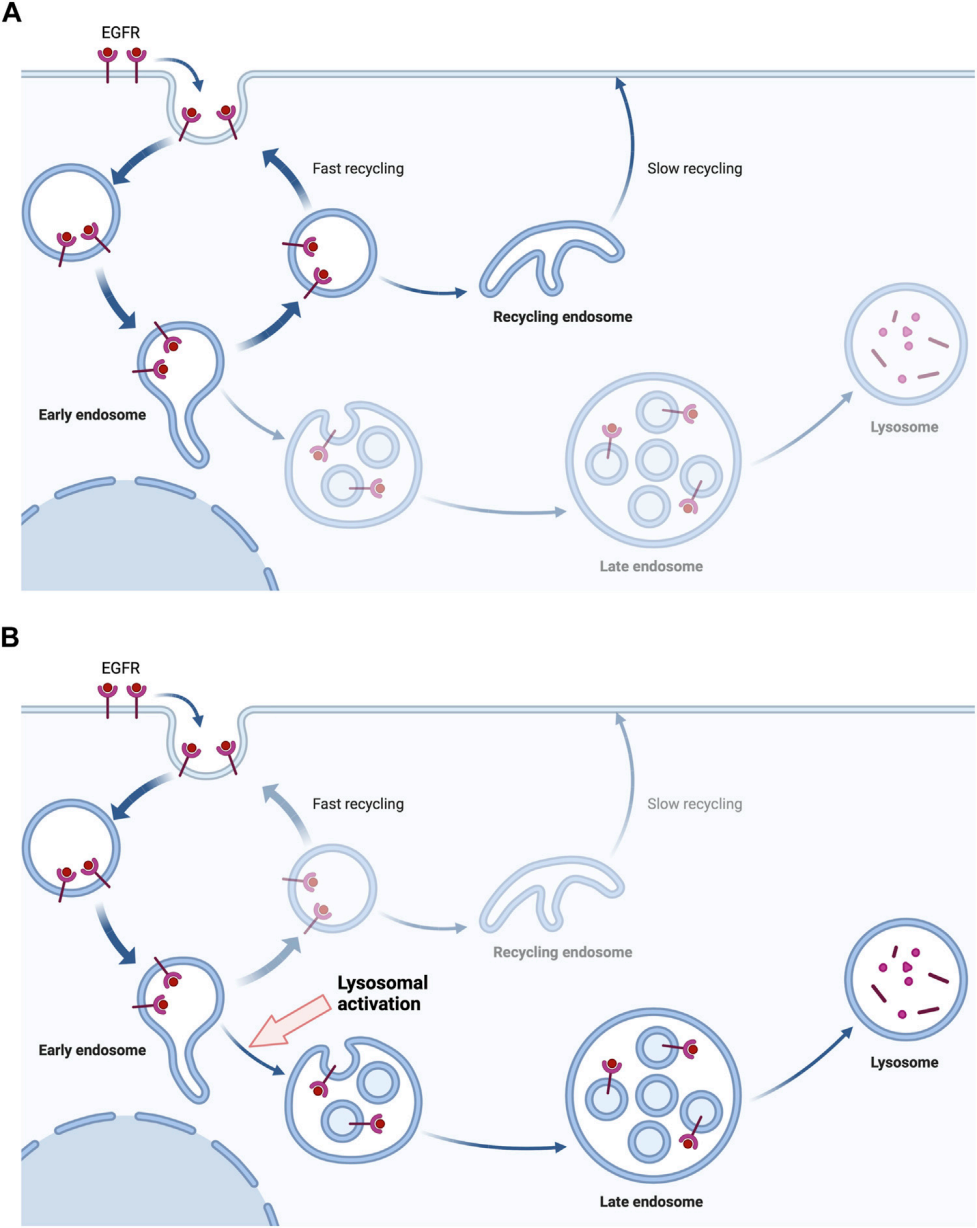
The lysosome activation drives the degradation of EGFR. The binding of EGF initiates the endocytosis of EGFR, inducing downstream signals. The endocytosed EGFR in endosomes is being recycled in tumor cells with EGFR mutation or overexpression **(A)**. However, the activation of lysosomes gives the drive force to the lysosomal degradation of endocytosed EGFR rather than the recycling **(B)**. Created with Biorender.com.

NF-κB activation *via* PI3K/Akt and MAPK pathways promotes not only the proliferation and metabolism but also inflammation signals **(** Figure 1 **)**. Enhanced IL-6 secretion is tightly correlated with the activation of ligand-mediated EGFR activation to promote the inflammatory tumor microenvironment of advanced-stage epithelial ovarian cancers (Alberti et al., 2012). In lung cancer, mucosa-associated lymphoid tissue 1 MALT1) cross-talks with NF-κB and STAT3 to develop an inflammatory tumor microenvironment (Pan et al., 2016). Interestingly, Erlotinib-resistant EGFR mutation in NSCLC enhanced NF-κB activity, while NF-κB inhibitors rescued the erlotinib sensitivity in resistant cells (Bivona et al., 2011). So, the inflammatory environment in tumor tissues could be mediated by EGFR *via* NF-κΒ and STAT3, and the blocking of EGFR signaling can suppress the promotion of tumor inflammations. This explains the importance of EGFR in tumor inflammations.

## 5 Therapies targeting EGFR and ErbB2

A number of tyrosine kinase inhibitors (TKIs) have been developed over the past 40 years, as well as still in preclinical and clinical studies (Yarden and Pines, 2012). These small molecules are considered the first choice in the treatment of solid tumors (Albanell and Gascon, 2005; Mendelsohn and Baselga, 2006). Currently, three generations of TKIs are being applied to target EGFR in practice and trials. Erlotinib and gefitinib are involved in the first-generation TKIs that reversibly competes with endogenous ATP to inhibit binding to the kinase domain (Yarden and Pines, 2012). The inhibition of ATP binding to the kinase domain leads to the suppression of tyrosine-phosphorylation, followed by the inhibition of downstream signals. These drugs are considered as the representative targeted therapy against solid tumors such as non-small-cell lung cancer (NSCLC) with kinase domain mutations, which show great response to EGFR TKIs. However, this is an exceptional example because it is well-known that a single TKI therapy usually shows poor therapeutic responses (Wheeler et al., 2010; Bria et al., 2011). The second-generation EGFR TKI includes afatinib, which irreversibly inhibits the kinase domain of EGFR and ErbB2 by binding to the free cysteine. Afatinib exhibits advantages over several platinum-based agents for the treatment of patients with EGFR mutations. However, most patients show inevitable development of acquired resistance after a median period of 10–14 months through several mechanisms (Sequist et al., 2011; Yang et al., 2012; Yu et al., 2013). Osimertinib is the third-generation TKI that targets mutations from first- and second-generation TKIs and shows high efficacy in first-line and second-line. However, resistance inevitably developed after the administration of osimertinib (Wu et al., 2020).

Antibody therapies targeting EGFR are another strategy to inactivate RTK signals. Cetuximab, panitumumab, nimotuzumab, and necitumumab prevent ligand binding and have been approved in several countries for colon cancer and head and neck cancer treatments (Mendelsohn and Baselga, 2006). In order that antibodies bind the receptors extracellularly, unlike the direct binding and inhibition of kinase, the antibodies prevent the dimerization of receptors in some cases (Mendelsohn and Baselga, 2006). The therapeutic effects of EGFR-targeting antibodies are relatively low compared to TKIs, showing only several months of survival in limited subtypes of patients (Siena et al., 2009). For example, colon cancer patients with wild-type KRAS in their tumors show good responses to EGFR-targeting antibodies (Siena et al., 2009). Recently, mixtures of new EGFR targeting antibodies like Sym004 (futuximab and modotuximab), a monoclonal antibody targeting multiple regions of EGFR extracellular domain like MM-151 and duligotuzumab as well as new monoclonal antibodies such as AMG595, anitumumab, depatuxizumab, GC118, humMR1, imagatuzumab, Mab A13, mafodotin, matuzumab, necitumumab, nimotuzumab, tomozotuximab, and zalutumumab are currently being studied (Cai et al., 2020).

## 6 Combination strategies targeting EGFR and ErbB2

In order to overcome the limitations of EGFR-TKIs and anti-EGFR antibodies, several combinations have been applied and are still under trial of TKIs and/or monoclonal antibodies with traditional chemotherapy, other inhibitors, and anti-angiogenic agents. The efficacy of the combination of EGFR-TKIs or monoclonal antibodies with chemotherapies shows remarkable outcomes, including in patients with head and neck tumors (Cai et al., 2020; Wu et al., 2020). However, the combination therapies also have limitations for tumors with EGFR-TKI-resistant patients in clinical trials in those with previous experience with TKIs (Laurila and Koivunen, 2015; Soria et al., 2015; Mok et al., 2017). Several combinations with platinum-based chemotherapies are under trial, but they still have a high possibility of showing resistance or other limitations (Asahina et al., 2021).

Unlike the combination of TKIs with classic chemotherapies, immune checkpoint inhibitors (ICIs) have shown promising therapeutic options in the second and third lines. EGFR mutations and EGFR TKI treatment had relevance with the increased PD-L1 levels, which suggests the combination of anti-PD-1/PD-K1 with TKIs may be synergistically effective to NSCLC (D’Incecco et al., 2015). However, the TATTON study revealed that Osimertinib treatment with durvalumab in patients with EGFR mutations shows higher risk of adverse effects (Oxnard et al., 2020). The CAUREL phase III clinical trial showed the effects of Osimertinib with or without nivolumab administration in T790M point mutation-bearing NSCLC patients who had previous TKI treatment. This study found patients with the combination of nivolumab and TKI showed markedly higher onset of interstitial pneumonitis than TKI single agent-treated patients (Oshima et al., 2018). Furthermore, other combination strategies of ICIs and TKIs also showed increased onset of adverse effects without significant improvement of efficacy (Oshima et al., 2018; Yang et al., 2018; Latif and Liu, 2019). Additionally, the combination of erlotinib with nivolumab showed tolerance and durable responses in EGFR mutation from clinical trials on TKI-treated NSCLC patients (Rizvi et al., 2016; Gettinger et al., 2018). In a nutshell, the combination therapy of TKIs and ICIs is in too early a stage, and further studies and assessments are required to treat cancer patients with EGFR overexpression or mutations. So, clinicians have to consider weighing the advantages and disadvantages, such as adverse reactions and therapeutic efficacy, when they make decisions to apply the agents. And therefore, more mechanistic studies of EGFR-mediated tumors are still going on to target the receptors and their roles in cancer.

## 7 Lysosomes

The lysosome is an acidic intracellular organelle containing hydrolases active in an acidic environment and specific membrane proteins. The lysosome lacks the mannose-6-phosphate receptor (M6PR), which is distinct from late-stage endosomes. These characteristics are shared by cell type-specific lysosomes such as melanosomes in melanocytes, delta granules in platelets, lamellar bodies in lung endothelial cells, lytic granules in lymphocytes, and other lysosomes in different cell types (Huizing et al., 2008).

Lysosomes exist in all animal cells except erythrocytes. Under the microscope, lysosomes are dense bodies in cytosol residing mostly in the perinuclear region. The shape of lysosomes ranges from spherical to tubular, and the size is different depending on the cell type from ∼ 1 μm through several μm. The shape, size, and number of lysosomes can be changed remarkably depending on the accumulation of internal undigested materials (Dingle and Shaw, 1979).

A phospholipid-bilayer surrounds lysosomes with 7–10 nm (Saftig et al., 2010). The difference between lysosomal membranes from other membranes is the high carbohydrate contents because 25 lysosomal membrane proteins are highly glycosylated (Lubke et al., 2009). The most profound membrane proteins are lysosome-associated membrane protein (LAMP)-1 and -2, lysosomal integral membrane protein (LIMP)-2, and CD63 (LAMP-3) (Eskelinen et al., 2003). The lysosomal structure is protected by glycocalyx formed by glycosylations at luminal domains of lysosomes from lysosomal hydrolases (Granger et al., 1990). In addition to the lysosomal membrane, lysosomes have intralysosomal membranes representing the primary membrane degradation site within lysosomes (Schulze et al., 2009). The intralysosomal membranes have abundant phospholipid bis (monoacylglycerol-phosphate (BMP), also known as lyso-bis-phosphatidic acid (LBPA). BMP is an exclusive marker of lysosomes and late-stage endosomes (Kobayashi et al., 1998).

Lysosomes enclose up to 600 μM of calcium content, a concentration similar to the classic calcium storage organelle, the endoplasmic reticulum (Bygrave and Benedetti, 1996; Christensen et al., 2002; Lloyd-Evans et al., 2008). Endosomal calcium has important roles in maintaining normal trafficking, recycling, and vesicular fusion (Lloyd-Evans and Platt, 2011). Lysosomal calcium release is obtained by nicotinic acid adenine dinucleotide phosphate (NAADP), a strong intracellular calcium-releasing secondary messenger, through its interaction with the two-pore channel (TPC) family (Churchill et al., 2002).

The finding of the coordinated lysosomal expression and regulation (CLEAR), a specific gene network of lysosomes, revealed the lysosomal biogenesis and regulation machinery. Many lysosomal genes interact with a CLEAR sequence (GTCACGTGAC) near the transcription initiation site (Sardiello et al., 2009). TFEB enters the nucleus, binds to the CLEAR site, and then induces lysosomal gene transcription. Nearly 500 direct TFEB target genes are known, including the lysosomal biogenesis and autophagy-related genes (Palmieri et al., 2011; Settembre et al., 2011). TFEB is the primary regulator of lysosomal function and orchestrates the function of the lysosomal gene network fulfilling the cellular needs of lysosomal degradations.

The lysosome is the major place of EGFR proteolysis, as well as other RTK molecules (Wiley and Burke, 2001). When EGF is bound to EGFR, the receptor is rapidly internalized by the kinase activity of the receptor and the specific motifs of the C-terminus. After the internalization of the receptor, EGFR is sorted into early endosomes and followed by late endosomes and lysosomes (Kil et al., 1999). RTKs share the common sorting and degradation paths, while the degree of regulation is different depending on the receptor. In the case of platelet-derived growth factor (PDGF) targets lysosome even without the presence of the specific ligand (Sorkin et al., 1991). This degradation process of RTKs, including EGFR can be a new therapeutic target against EGFR-mediated tumor cells. Since the regulation of lysosomal activity is distinct from the inhibition of the ligand-binding or kinase inhibition, lysosomal activation can be the new therapeutic approach as a combination therapy with EGFR antibodies or TKIs. There are additional roles of endosomes and lysosomes in immune cells. Lysosomes process antigens in antigen-presenting cells (APCs) for host-defense mechanisms. Granulocytes and monocytic cells degrade endocytosed or phagocytosed pathogens or tumor antigens. They also present processed antigens on MHCs for further process by T cells (Watts, 2022).

## 8 Conclusion and perspectives

**FIGURE 2:**
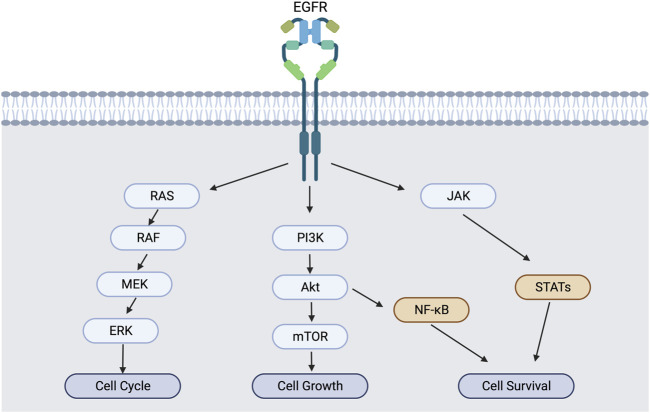
EGFR activation and downstream signaling cascade. EGFR activation induces not only MAPK/PI3K pathways, but also NF-κB and STAT pathways for cell survival and inflammation.

The combination of anti-cancer agents is inevitable as the continuous mutations and overexpression is making the tumors more resistant to single-agent therapies. The limitations and adverse effects are current obstacles in EGFR targeting therapies. However, this will be a powerful strategy to overcome a huge portion of current cancers. In a recent study, lysosome activation leads to the vigorous degradation of RTKs, including EGFR (Hong et al., 2019). As RTKs are given with the driving force to be degraded rather than recycled, the downstream signal of EGFR will inevitably be decreased. This may be one of the new therapeutic strategies to target EGFR family molecules in combination with TKIs or anti-EGFR antibodies **(** Figure 2 **)**. As lysosomal degradation is the further later step in the pathway of EGFR signals, targeting lysosomes may show a good synergistic effect with TKIs and anti-EGFR antibodies. Experimentally, the synergistic effect of lysosomal activation and an anti-EGFR monoclonal antibody has been observed (Hong et al., 2019), so further study to discover a specific lysosome-activating agent or gene-therapy targeting tumor cells may be the next field to overcome EGFR-mediated tumors. Currently, lysosome-activating agents are not available for both therapeutic and experimental usages and we believe the development of specific agents to target lysosomes will be much helpful for future cancer treatments.
